# Tracking excess of maternal deaths associated with COVID-19 in Brazil: a nationwide analysis

**DOI:** 10.1186/s12884-022-05338-y

**Published:** 2023-01-12

**Authors:** Raphael Mendonça Guimarães, Lenice Gnocchi Costa Reis, Maria Auxiliadora de Souza Mendes Gomes, Cynthia Magluta, Carlos Machado de Freitas, Margareth Chrisostomo Portela

**Affiliations:** 1 Fundação Oswaldo Cruz, COVID-19 Observatory, Avenida Brasil, 4365, Manguinhos, Rio de Janeiro, RJ 21041-960 Brazil; 2 Fundação Oswaldo Cruz, Fernandes Figueira National Institute for Women, Children and Youth, Avenida Rui Barbosa, 716, Flamengo, Rio de Janeiro, RJ Brazil

**Keywords:** COVID-19, SARS-CoV2, Maternal mortality, Brazil

## Abstract

**Background:**

The COVID-19 pandemic brought a new challenge to maternal mortality in Brazil. Throughout 2020, Brazil registered 549 maternal deaths, mainly in second and third-trimester pregnant women. The objective of this study was to estimate the excess maternal deaths in Brazil caused directly and indirectly by Covid-19 in the year 2020. In addition, we sought to identify clinical, social and health care factors associated with the direct maternal deaths caused by Covid-19.

**Methods:**

We performed nationwide analyses based on data from the Mortality Information System (SIM) for general and maternal deaths and the Influenza Epidemiological Surveillance System (SIVEP-Influenza) for estimates of female and maternal deaths due to COVID-19. Two distinct techniques were adopted. First, we describe maternal deaths directly caused by covid-19 and compare them with the historical series of deaths from covid-19 among women of childbearing age (15 to 49 years). Next, we estimated the total excess maternal mortality. Then, we calculated odds ratios for symptoms, comorbidities, social determination proxies and hospital care aspects between COVID-19 maternal deaths and deaths of women of childbearing age who were not pregnant or no maternal deaths. We chose women of childbearing age (15 to 49 years) as a reference because sex and age introduce differentials in the risk of COVID-19 death.

**Results:**

Most maternal deaths occurred during pregnancy compared to postpartum deaths month by month in 2020 (μ = 59.8%, SD = 14.3%). The excess maternal mortality in 2020 in Brazil was 1.40 (95% CI 1.35–1.46). Even considering excess mortality due to COVID-19 for the childbearing age female population (MMR 1.14; 95% CI 1.13–1.15), maternal mortality exceeded the expected number. The odds of being a black woman, living in a rural area and being hospitalized outside the residence municipality among maternal deaths were 44, 61 and 28% higher than the control group. Odds of hospitalization (OR 4.37; 95% CI 3.39–5.37), ICU admission (OR 1.73; 95% CI 1.50–1.98) and invasive ventilatory support use (OR 1.64; CI 95% 1.42–1.86) among maternal deaths were higher than in the control group.

**Conclusions:**

There was excess maternal mortality in 2020 in Brazil. Even with adjustment for the expected excess mortality from Covid-19 in women of childbearing age, the number of maternal deaths exceeds expectations, suggesting that there were deaths among pregnant and postpartum women indirectly caused by the pandemic, compromising access to prenatal care., adequate childbirth and puerperium.

## Background

Overall Brazil’s maternal mortality ratio (MMR) ranged from 60 to 65 deaths per 100,000 live births between 2010 and 2017 [1]. The country did not accomplish Millennium Development Goal 5 (MDG), established at a reduction of 75% of MMR from 1990 to 2015. In the Sustainable Development Goals (SDG) for 2030, a new MMR goal of 70 deaths per 100,000 live births was set. However, the COVID-19 pandemic brought new challenges. COVID-19 was declared a public health emergency of international concern (PHEIC) on January 30 and a pandemic on March 11, 2020. Pregnant and postpartum women represented a COVID-19 fatality rate of 7.2%, almost three times as high as the 2.6% in the general population in the country. Pregnant women had not been identified as being particularly vulnerable before the time that COVID-19 was still majorly affecting high-income countries.

Studies have addressed the impact of COVID-19 on pregnancy, childbirth and the puerperium and whether that period changes the natural history of COVID-19 [2, 3]. COVID-19 in pregnant women has been associated with higher cesarean section rates and mortality [4]. The effects on maternal outcomes have yet to be understood, however. We already know that pregnant women’s risks include relative immunodeficiency associated with maternal physiological adaptations and an intense inflammatory reaction due to infection. In addition, pregnant women can suffer from multiple organ failure and comorbidities play a significant role [5].

Maternal mortality is also affected by the quality of maternity care, which involves access, availability of necessary resources and acceptable practices for antenatal care, childbirth and puerperium [6]. The impact of the pandemic on the provision of regular antenatal care and the worsening lack of ICU beds for obstetrics created additional difficulties in the clinical management of women with high-risk pregnancies, regardless of COVID-19 infection [7]. Thus, the objective of this study was to verify excess maternal deaths in Brazil caused directly and indirectly by Covid-19 in the year 2020. In addition, we sought to identify clinical, social and health care factors associated with direct maternal deaths caused by Covid-19.

## Methods

We performed nationwide analyses based on data from the Mortality Information System (SIM) for maternal deaths. To analyze deaths related to Covid-19, we also obtained data from deaths due to Covid-19 among women of childbearing age from the Influenza Epidemiological Surveillance System (SIVEP-Influenza).

The methodological approach adopted two distinct techniques. First, we describe maternal deaths directly caused by covid-19 and compare them with the historical series of deaths from covid-19 among women of childbearing age (15 to 49 years). Next, we estimated the total excess maternal mortality. Next, we defined social and clinical characteristics in the second step and compared maternal deaths (pregnant and postpartum females) to childbearing age from COVID-19. Like the previous step, we chose women of female deaths among women of childbearing age (15 to 49 years) since sex and age introduce differentials in the risk of COVID-19 death..

- Excess Mortality

We obtained monthly counts of maternal deaths between 2015 and 2019. The definition of maternal deaths includes deaths among pregnant women or women who died up to 6 weeks after childbirth. From the linear trend of this period, we estimated the number of maternal deaths expected for the year 2020. Then, we compared the number of predicted maternal deaths and the observed numbers in 2020. The comparison allowed calculation of excess mortality, represented by the difference between the observed and expected values [8]. Furthermore, the pandemic caused an excess of general mortality [9]. We could thus verify whether the excess of maternal mortality differed from the excess of general mortality (considering only women of reproductive age). We calculated the excess of general mortality and used this excess as a correction factor for the expected value of maternal mortality. In this way, we created two analysis scenarios:

- The one whose expected value of maternal death considers the trend of previous years;
- The one whose expected value of maternal death considers the trend of previous years, corrected for the excess general mortality of women of childbearing age (10–49 years).

In addition, we estimated the 2020 excess maternal mortality and its 95% confidence interval using a Poisson model with robust variance.

b) Mortality Odds Ratio

The database we used for the study includes hospitalizations for severe acute respiratory syndromes. We intended to compare hospitalizations and deaths from Covid-19 in women of childbearing age who were not pregnant or who had recently given birth (within 6 weeks) and maternal deaths from Covid-19. As they are mutually exclusive groups (with or without maternal death status), we used mortality odds ratios (MOR). This measure is helpful when death data are available, but the population at risk is unknown [10]. Considering that only deaths were considered, not allowing direct risk estimates, we performed comparisons based on mortality odds ratios (MOR) and 95% confidence intervals [11].

We compared female deaths and maternal deaths whose underlying cause was COVID-19 based on clinical symptoms (fever, cough, throat Pain, dyspnea, respiratory distress, oxygen saturation < 95%, diarrhea and vomiting), comorbidities (cardiovascular disease, hematological diseases, liver diseases, asthma, diabetes, neuropathies, lung diseases, immunodepression conditions, kidney diseases and obesity), hospitalization circumstances (hospital admission, ICU admission, invasive respiratory support utilization; non-invasive respiratory support utilization) and social determinants proxy variables (race, area of residence – rural or urban, and out-of-city hospitalization), all included on the SARS notification form.

We performed statistical analysis using the R program, version 4.1.2.

## Results

**Fig. 1:**
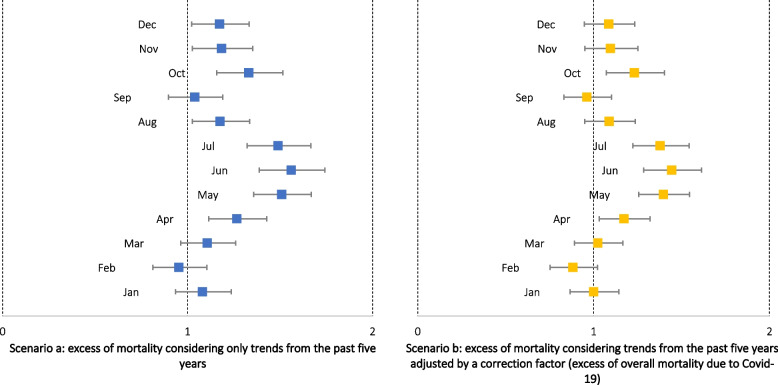
Excess maternal mortality per calendar month according to expected death scenarios. Brazil, 2020. Source: SIVEP-Gripe, 2021

In 2020, 549 COVID-19 deaths in pregnant and postpartum women were reported with a weekly average of 12.1 deaths. Excess maternal mortality in 2020 in Brazil was 1.40 (95% CI 1.35–1.46). Comparing the observed maternal deaths with expected numbers obtained by the trend of the previous 5 years (Scenario 1, Fig. 1a), we found a statistically significant excess of mortality from April to August and between October and December 2020. In June, the peak of the first pandemic wave, there was an excess of 56% of maternal deaths (SMR = 1.56, CI 95% 1.39–1.74) compared to the expected value for the same month.

In another scenario, we corrected the predicted number by a factor estimated from the overall excess mortality in women of childbearing age in 2020 (Scenario 2, Fig. 1b). Even with this increase, we obtained a statistically significant excess of mortality for the months from April to July and October. The month with the highest excess was July (SMR = 1.44, CI 95% 1.28–1.61). It reinforces the suggestion that even with an increase in general female mortality due to COVID-19, pregnant and postpartum women were at higher risk of excess of maternal mortality than general female mortality.

We found that 70% of the number of women who died were attributable to Covid-19. Hypothetically, the other numbers (30%) were due to other health issues, including preventable deaths related to barriers faced by women to timely adequate maternity care and the worsening performance and quality of care. Maternal and female deaths due to COVID-19 were compared by clinical inpatient care and social variables using MOR, suggesting no statistically significant differences among maternal deaths compared to women of childbearing age (Table 1).

**Table 1:** Maternal and female COVID-19 Mortality Odds Ratio (MOR) per social determinant proxy, clinical and inpatient care variables. Brazil, 2020

Dimension of Risk Factors	MOR	CI 95%
**Social Determinant Proxies**		
Black vs. Non-Black People	1.44	1.22 to 1.66
Rural vs. Urban/Periurban Place	1.61	1.24 to 1.98
Out-of-City Hospitalization	1.28	1.09 to 1.48
**Symptoms**		
Fever	0,91	0.69 to 1.14
Cough	1.15	0.90 to 1.40
Throat Pain	1.08	0.84 to 1.33
Dyspnea	0.75	0.48 to 1.03
Respiratory Distress	1.00	0.75 to 1.25
Oxygen saturation < 95%	0.82	0.82 to 1.06
Diarrhea	0.78	0.46 to 1.10
Vomiting	0.68	0.32 to 1.04
**Comorbidities**		
Overall	0.50	0.25 to 0.80
Cardiovascular Disease	0.36	0.09 to 0.66
Hematological Diseases	0.88	0.15 to 1.60
Liver Diseases	1.33	0.68 to 1.99
Asthma	0.69	0.21 to 1.18
Diabetes	0.32	0.10 to 0.59
Neuropathies	0.64	0.08 to 1.21
Lung diseases	1.02	0.51 to 1.55
Immunodepression conditions	0.64	0.25 to 1.04
Kidney Diseases	0.61	0.18 to 1.03
Obesity	0.53	0.21 to 0.85
**Hospital Care**		
Hospital Admission	4.37	3.39 to 5.37
ICU admission	1.73	1.50 to 1.98
Invasive Respiratory Support (yes vs. no)	1.64	1.42 to 1.86
Non-Invasive Respiratory Support (yes vs. no)	0.57	0.32 to 0.82

Source: SIVEP-Gripe

Regarding comorbidities recorded in the SIVEP-Influenza, overall occurrence was lower among maternal deaths than among non-maternal female deaths due to COVID-19 (MOR = 0.50; 95%CI 0.25–0.80). The reason, however, for this difference was due to three co-morbidities: cardiovascular diseases (MOR = 0.36; 95% CI 0.09–0.66), diabetes mellitus (MOR = 0.32; 95% CI 0.10–0.59) and obesity (MOR = 0.53; 95% CI 0.21–0.85). Due to their prevalence, the weight of these comorbidities is high. Maternal deaths had a lower risk of occurrence than deaths of women of childbearing age.

Regarding social variables, risks of maternal death were higher among black women (MOR = 1.44, CI 95% 1.22–1.66), women living in rural areas (MOR = 1.61, CI 95% 1.24–1.98) and women assisted outside their cities of origin (MOR = 1.28, CI 95% 1.09–1.48). Finally, concerning inpatient care variables, women who died during pregnancy and childbirth were more likely to have undergone clinical hospitalization (MOR = 4.37, 95% CI 3.39–5.37), ICU hospitalization (MOR = 1.73, 95% CI 1.50–1.98) and to have been submitted to invasive ventilatory support (MOR = 1.64, 95% CI 1.42–1.86).

**Fig. 2:**
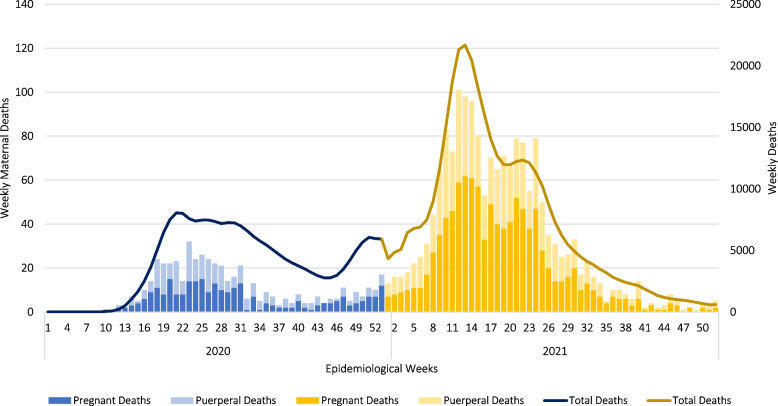
Overall and maternal mortality time series. Brazil, 2020–2021. Source: SIVEP-Gripe, 2021

Finally, we could report analysis for the year 2020. There has been a rise in mortality in Brazil since April 2020. Between the beginning of July and the end of August, the daily number of deaths was stabilized at a high level with approximately 1000 deaths daily. Then, there was a gradual drop in deaths until the end of October, when daily deaths reached around 300. After that, there was a new resumption of growth in the curve. At the end of December 2020, the average number of daily deaths was 700, with a cumulative total of 180,000 deaths in the country since the beginning of the pandemic. Variations in maternal mortality caused by Covid-19 followed the same trend in overall Covid-19 deaths (Fig. 2). Although we did not analyze the year 2021, due to lack of information on country-wide general mortality, we chose to present deaths by Covid-19. From this data, we could check that that the number of maternal deaths from Covid-19 in 2021 (1757) exceeded 2020 by 323%.

## Discussion

Maternal mortality is a largely preventable event (> 90%). It is directly associated with increasing poverty and starvation [12]. High maternal mortality ratios (MMRs) are mainly concentrated in countries with a peripheral economy, and these reveal severe violations of women’s rights to health.

Since mid-2020, publications on the deaths of pregnant and postpartum women in Brazil have alerted us to prepare and organize a complete healthcare network [13]. It focused on ensuring timely access and adequacy of clinical practices because of the specificities of Covid-19 in the pregnancy-puerperal cycle. Variables related to hospital care and social determinants of health were associated with increased odds of maternal mortality.

Social inequality plays a central role in explaining excess maternal deaths. Differences among pregnant women’s profiles in Brazil and other countries can be explained [14]. There is a high occurrence of comorbidities in the country whose etiology involves inflammatory processes, which are risk factors for complications from Covid-19, such as obesity, hypertension, diabetes and vasculopathy [15].

Conversely, these comorbidities were less associated with maternal deaths. These findings were previously described by Scheler et al. [16]. This study identified that comorbidity was associated with increased fatality rates for both groups but higher in the non-obstetric group (22.8% vs. 13.3%). We believe that the leading chronic conditions for which we identified decreased risks of maternal mortality, indicate different interventions in prenatal care. Pregnant women with these comorbidities tend to have better prior monitoring because of their high risks and may be more sensitive to changes with early intervention. Timely access to prenatal care thus is a strong determinant of controlling previous chronic conditions, which determines women’s prognosis.

Social determinants of health strongly influence these chronic conditions but are even more associated with access to health care. Social, economic and health policies would benefit from considering this contextual effect [17]. Therefore, racial, geographic and socioeconomic disparities require special attention [18]. Women with COVID-19 and deaths in the obstetric population had a heterogeneous geographical distribution. Municipalities with high socioeconomic dissimilarities showed higher MMRs than areas with better social and infrastructure indicators [18].

In low- and middle-income, as opposed to high-income countries, high birth rates and limited resources for healthcare contribute to increased risks of maternal death due to COVID-19 [19]. Brazil, however, has had a declining birth rate since the 1970s and poor quality prenatal and maternity care has a more powerful explanatory weight. Timely access to adequate maternity care is essential for women’s safety and quality of care [20]. Delay in receiving care is associated with adverse maternal outcomes. Thaddeus & Maine [21] developed the “Three Delays” model to assess access to maternity care, broken down into three phases: (1) delay in the decision to seek health care; (2) delay in identifying and reaching appropriate health facilities; and (3) delay in receiving appropriate care at the right time. This model is used today to explain severe maternal morbidities and deaths.

In Brazil, barriers to access maternity care with specialized, complex conditions and inadequate monitoring of obstetric complications persist [22]. They occur despite the warning of the CDC that COVID-19 increases the risk of pregnant and postpartum women to develop more severe forms, requiring hospitalization, intensive care and mechanical ventilation [23]. The number of maternal deaths in 2020 was impressive, but the numbers were more than three times higher in 2021 [24].

The pandemic also aggravated the difficulties in accessing maternity care across the country. Excess maternal mortality may be directly or indirectly related to this increase. Similar results had already been pointed out previously on a sub-national scale in Brazil [25]. The assumption of maternal deaths because of COVID-19 and unequal access to health care is also corroborated by Obstetric Observatory BRAZIL - COVID-19, which analyzes nationwide public databases to provide an interactive scientific monitoring platform-based analysis, disseminating relevant information regarding maternal and child health in the country [26].

The situation in Brazil demonstrates the importance of national leadership in confronting a pandemic. It is even more important to recognize the need for long-term global care to improve local public health [27]. High MMRs suggest the failure of the Brazilian health system. A solution requires the international community’s involvement since it affects global development. This scenario does not repeat itself in other LMICs, prolonging the pandemic’s impact on all [28]. For this reason, analysis of excess maternal mortality is a call for action at this point in the pandemic.

Our analysis has limitations. Total mortality data represent data available at the time of the study. Data, however, can change due to updates over the next few months. The maternal death pattern, however, is already worrying in the current scenario. Another limitation concerns low testing in Brazil. It prevents us from precisely knowing the numbers of COVID-19-infected pregnant and postpartum women. This information does not compromise the estimated calculation we use, however. Mortality Odds Ratios are precise in these circumstances, where the population base is unknown. We assume deaths in the general population (in our case: the childbearing age female population) to estimate the odds of factors we considered to be related to maternal mortality. We also know there is some imbalance between the age group in which most women with pregnancies (between 20 and 29 years old) are concentrated and the group with the highest prevalence of comorbidities (40 to 49 years old). However, we performed our analyses disregarding pregnancies’ order and we believe this minimized potential selection bias.

The COVID-19 pandemic may represent a significant immediate obstacle to Brazil’s achievement of SDGs by 2030. Excess maternal mortality and the considerable increase in women with near-misses caused by COVID-19, directly or indirectly, have placed the country in a precarious situation. Vaccination against Covid-19 started in Brazil in January 2021. Thus, it had no implications for our analysis. On the contrary, our evidence reinforces the need for expanded immunization in this group, including maintaining the calendar as a priority in vaccine booster doses.

To sum up, weWe should mention that this study concerns the very beginning of the pandemic when pregnancy was not yet identified as a clear risk factor and only the wild-type variant of Sars-Cov-2 was endemic. As soon as 2021 mortality data are available for Brazil, it is important to extend the present analysis to the entire period. Moreover, it is necessary to combine non-pharmacological measures and vaccination. We need to strengthen maternal health care from access to antenatal care to regulating obstetric ICU beds. Antenatal consultations must be qualified, encouraging physical distance measures. We also need to screen pregnant women for respiratory symptoms, distribute good quality masks, adopt universal testing on admission to maternity hospitals with molecular tests (RT-PCR) and provide obstetric care in hospital units with access to ICU beds for women with moderate and severe disease.

## Data Availability

The datasets generated and/or analyzed during the current study are available in the Ministry of Health of Brazil, SRAG 2020 - Severe Acute Respiratory Syndrome Database - including data from COVID-19 -repository, available at https://opendatasus.saude.gov.br/dataset/bd-srag-2020
